# Bronchoscopic Intra-Pleural Instillation of Fibrin Glue and Autologous Blood to Manage Persistent Air Leaks after Lung Resection

**DOI:** 10.3390/jcm11071934

**Published:** 2022-03-30

**Authors:** Giorgio Maria Ferraroli, Gianluca Perroni, Veronica Maria Giudici, Alberto Antonicelli, Hiran Chrishantha Fernando, Vincenzo Ambrogi, Marco Alloisio, Emanuele Voulaz, Edoardo Bottoni, Maurizio Valentino Infante, Alberto Testori

**Affiliations:** 1 Division of Thoracic Surgery, IRCCS Humanitas Research Hospital, 20089 Milan, Italy; giorgio_maria.ferraroli@humanitas.it (G.M.F.); veronica.giudici@humanitas.it (V.M.G.); marco.alloisio@humanitas.it (M.A.); emanuele.voulaz@humanitas.it (E.V.); edoardo.bottoni@humanitas.it (E.B.); alberto.testori@humanitas.it (A.T.); 2 Department of Thoracic Surgery, Tor Vergata University, 00133 Rome, Italy; ambrogi@uniroma2.it; 3 Division of Thoracic Surgery, IRCCS San Raffaele Hospital, 20132 Milan, Italy; antonicelli.alberto@gmail.com; 4 Department of Cardiothoracic Surgery, Allegheny General Hospital, Pittsburgh, PA 15212, USA; chrishf@mac.com; 5 Department of Biomedical Sciences, Humanitas University, 20090 Milan, Italy; 6 Department of Thoracic Surgery, University and Hospital Trust of Verona, 37126 Verona, Italy; mauriziovalentino.infante@univr.it

**Keywords:** flexible thoracoscopy, persistent air leak, prolonged air leak, fibrin glue, blood patch, bronchoscopy, thoracotomy, thoracic surgery, lung resection, prolonged length of stay

## Abstract

Background: Persistent air leak is a common complication after lung resection causing prolonged length of stay and increased healthcare costs. Surgical intervention can be an option, but other more conservative approaches should be considered first. Here, we describe the use of flexible bronchoscopy to apply fibrin glue and autologous blood sequentially to the damaged lung. We named the technique “flexible thoracoscopy”. Methods: Medical records from patients with persistent air leaks after lung resection were collected retrospectively. Depending on the type of aerostasis that was performed, two groups were created: flexible thoracoscopy and surgery (thoracotomy). Flexible thoracoscopy was introduced at our institution in 2013. We entered the pleural space with a bronchoscope following the same surgical pathway that was used for tube thoracostomy. Perioperative characteristics and outcomes were analyzed using R software (ver. 3.4.4). Results: From 1997 to 2021, a total of 23 patients required an intervention for persistent air leaks. Aerostasis was performed via flexible thoracoscopy in seventeen patients (69%) and via thoracotomy in six patients (31%). The median age was 70 years (22–82). Twenty patients were males (87%). There was no difference in age, sex distribution, BMI, comorbidities and FEV1%. An ASA score of 3 was more represented in the flexible thoracoscopy group; however, no evidence of a difference was found when compared to the thoracotomy group (*p* = 0.124). Length of in-hospital stay and chest tube duration was also similar between groups (*p* = 1 and *p* = 0.68, respectively). Conclusions: Aerostasis achieved either by flexible thoracoscopy or by thoracotomy showed similar results. We believe that flexible thoracoscopy could be a valid alternative to facilitate minimally invasive treatments for persistent air leaks. Further studies are needed to confirm these results.

## 1. Introduction

Persistent air leak (PAL) is one of the most common postoperative complications after elective lung resection (10.4% [1]). PAL can be caused by either an alveolar-pleural fistula (APF) or bronchopleural fistula (BPF). PAL is arbitrarily defined as an air leak lasting for more than five days following lung resection [2] and it is associated with an increased risk of postoperative pneumonia, empyema and deep vein thrombosis [3,4]. PAL is also associated with prolonged length of stay (LOS), increased healthcare costs and a greater risk of mortality [5,6].

Several different strategies can be used to manage PALs. They range from an endoscopic approach up to open surgery. Bronchoscopic placement of either sealants or uni-directional valves represents the least invasive intervention in the spectrum of aerostatic treatments. Autologous blood patch pleurodesis is also a valid alternative. However, none of these treatments allows direct visualization of the active source of air leakage (5). On the opposite side of the spectrum, surgical aerostasis attempted either by thoracoscopy or thoracotomy can lead to the visual identification of the tissue defect, hence, to a targeted treatment. Nonetheless, surgical aerostasis is an invasive approach carrying surgical risks and often requires general anesthesia and one-lung ventilation.

Invasive and minimally-invasive approaches for aerostasis showed varying results with no clear superiority of one solution over the other [7]. Direct visualization with quick and effective management of a complication, as we know it, is the ideal framework for any surgeon. Since this is usually achieved surgically, though, it also means a more invasive approach for the patient. Going less invasive, on the other hand, means losing that direct visual feedback on the air leakage which, in turn, can decrease the accuracy of the aerostatic maneuver. We sought to investigate a technique that would combine direct visualization with minimal trauma.

The first report on using a flexible bronchoscope (FB) as an alternative to a rigid thoracoscope for pleural space visualization was described by Senno et al. in 1974 [8]. He demonstrated that the insertion of a FB under local anesthesia through a chest incision was possible for the diagnosis of pleural diseases, e.g., primary and secondary pleural neoplasms. Gwin et al. supported the diagnostic role of FB by successfully performing pleural biopsies on visible pleural nodules [9].

Later, Tsukamoto et al. used a similar approach in 15 patients with spontaneous pneumothorax. They improved the visualization of the damaged parenchyma by passing the bronchoscope through the irrigation sheath of a hysteroscope. Air leaks were stopped safely and effectively after bleb coagulation or injection of a sealant through the operative channel [10].

A semi-rigid thoracoscope is a hybrid instrument that can be used for the diagnostic evaluation of pleural effusions by interventional pneumologists. It combines the maneuverability of a thoracoscope with the flexibility of a bronchoscope. To the best of our knowledge, there is no evidence of the use of this instrument to treat PALs.

Among all sealants commercially available, fibrin glue is a promising option due to its high efficacy rates and safety [11].

At our institution, aerostasis for PALs after lung resection used to be executed by thoracotomy. However, this approach requires a reintervention under general anesthesia carrying an additional risk for postoperative complications, such as atelectasis, pneumonia, respiratory failure, cardiac arrhythmias and ischemia [12,13].

In 2013, we started using FB to instill fibrin glue and autologous blood directly on leaky parenchyma by simply following the same surgical pathway that was used for tube thoracostomy. Our goal was indeed to leave thoracotomy as the last option.

To avoid confusion with other procedures, e.g., rigid thoracoscopy or semi-rigid thoracoscopy we named this technique flexible thoracoscopy (FT).

This study aimed to describe the efficacy of FT in treating PALs following lung resection. The results were compared with the medical records from a previous group of patients who received thoracotomy at the same institution (Humanitas Research Hospital). FT was performed by one surgeon only (G.M.F.).

## 2. Materials and Methods

### 2.1. Selection Criteria

We retrospectively collected medical records of patients over 18 years of age with PAL after lung resection from 1997 to 2020. We selected patients with PAL that did not resolve within 10 days from the surgical procedure. These patients did not tolerate either being placed on a water-seal or on a Heimlich valve. Subcutaneous emphysema and lung collapse at chest X-ray were observed. Patients with benign diseases, severe heart disease, renal impairment and an ASA score of 4 or higher were excluded from the analysis.

Then, we divided the sample group according to the method used to treat PAL. In other words, those patients who underwent FT (from 2013 to 2020) were compared with those who received thoracotomy (from 1997 to 2012, namely “the historical group”). In the case of anatomical lung resection, the possibility of a broncho-pleural fistula was verified and excluded in both groups by checking the integrity of the bronchial stump with FB. This study was approved by our ethical review board (cod. FLEX1).

### 2.2. Procedure

FT took place in the bronchoscopy suite. The patient was in a semi decubitus position with the ipsilateral arm raised over the head. Vital parameters were continuously monitored. Patient cooperation was possible throughout the entire procedure as only local anesthesia was needed (15–30 mL of lidocaine 2%).

A water reservoir along with alternate chest tube clamping was used to verify which of the two chest tubes was draining the greater amount of air. Skin disinfection was achieved with iodopovidone 10%. A sterile field was prepared. The targeted chest tube was then removed and the residual thoracostomy was used to introduce the flexible bronchoscope (2.8 mm channel size, Fujifilm Holdings Corporation, Tokyo, Japan) and gain access to the pleural cavity. To reduce the risk for germ mobilization from the thoracostomy into the pleural cavity while inserting the bronchoscope, iodopovidone 10% was also applied around its shaft.

The lung parenchyma was inspected for any possible area of damage. A thorough inspection was possible thanks to the partial lung collapse caused by the concurrent PAL. To facilitate the identification of the active source of PAL, saline solution was instilled in the pleural space and the patient was asked to cough. Four to 8 mL of fibrin glue (Tisseel, Baxter Healthcare, Deerfield, IL, USA) was applied to the targeted lesion via a 1.7 mm flexible polyurethane catheter (Duplocath, Baxter Healthcare, Deerfield, IL, USA) deployed through the working channel of the bronchoscope. Since such a catheter is made by two channels, the fibrin glue compounds were kept separate on their way down through the bronchoscope, and therefore, clogging could not occur. To further reduce the risk of clogging, the fibrin glue was instilled as quickly as possible.

Ten milliliters of autologous blood were then collected from a peripheral vein and applied as-is on the same targeted lesion through the operative channel of the bronchoscope. Lavage with saline solution was then carried out to test for further air leakage. Once the desired aerostasis was achieved, the bronchoscope was removed and a new chest tube (24 or 22 Fr) was placed via the same thoracostomy used to perform FT.

### 2.3. Post Procedure Management

Patients were monitored for direct and indirect signs of air leaks, e.g., looking for bubbles in a conventional, wet-seal, chest drainage canister and taking a chest X-ray (CXR) within 24 h. Air leaks were evaluated according to the scale proposed by Sang et al. in 2017 and the CXR was repeated every other day [14]. Discharge was accepted with no to minimal evidence of air leak (grade 0–2) and no radiographic worsening: if no air leak was observed, both chest tubes were removed; Otherwise, one chest tube would be removed hence sending patients home with a single chest tube attached to a Heimlich valve draining into a plastic bag for qualitative air monitoring. Patients were instructed to return to the hospital for an outpatient visit when the bag stopped inflating with air. The remaining chest tube was then connected to a traditional, wet-seal, chest drainage canister for 30 min with no externally applied suction. If no bubbles were observed, then the chest tube was removed.

### 2.4. Statistical Analysis

Data were described as numbers and percentages or as median and range. Differences in the distribution of categorical and continuous variables were tested using the Χ^2^ and the Wilcoxon t-tests, respectively. All evaluations were explorative in nature. A *p*-value of 0.05 was considered of potential statistical interest. All analyses were carried out with R software (version 3.4.4) (R Foundation for Statistical Computing, Vienna, Austria).

## 3. Results

A total of 23 patients met the inclusion criteria. To manage PAL, 17 patients received FT while six had a thoracotomy (historical group). Clinicopathological characteristics are shown in Table 1. The median age at surgery was 70 years (22–82). Twenty patients (87%) were male. Ten patients (43.5%) never smoked, seven (30.4%) quit and another six (26.1%) were active smokers. Lung resection was performed by thoracotomy in 18 patients (78.3%). Tumor histology (Table 1) showed adenocarcinoma in 12 patients (52.2%), squamous cell carcinoma in four (17.4%) and other types in seven patients (30.4%). Five patients (21.7%) were discharged home with a Heimlich valve.

When baseline characteristics were analyzed (Table 2), the FT group showed a median age of 71 years (47–82), a BMI of 24 (19–27) and an FEV1% of 83 (49–96). The median age was higher and an ASA score of 3 was more represented, however, no evidence of a difference was found when compared to the historical group (*p* = 0.076 and *p* = 124, respectively). Sex distribution (*p* = 0.54), smoking status (*p* = 0.13) and comorbidities (*p* = 0.62) were similar between groups. Lung resection had been performed by thoracotomy in 14 patients (82%), involving the right side in 14 patients (82%) with an upper lobe resection in 12 (71%). These results did not statistically differ from those obtained in the historical group.

When postoperative characteristics and outcomes of the FT group were analyzed (Table 3), the time between lung resection and intervention for PAL was 13 days (7–41). The chest tube duration from the time of aerostasis was 10 days (2–31). No evidence of a difference was found when these results were compared to those from the historical group (Table 3). The length of in-hospital stay (*p* = 1) was also similar.

## 4. Discussion

Persistent air leak remains a challenge for thoracic surgeons [7]. It is one of the most common complications after lung resection and it is associated with increased hospital length of stay, higher morbidity and mortality, increased pain and discomfort. All this means greater health care costs. We ourselves were not able to manage our patients conservatively due to the onset of subcutaneous emphysema and radiographic worsening of the pneumothorax.

Our results show that aerostasis achieved by FT can be as effective as the one performed by thoracotomy.

FT makes it possible to directly inspect the pleural cavity without causing the trauma of a thoracotomy. Furthermore, structures inside the pleural space can be magnified by FT, and therefore, even minimal alveolar-pleural fistula can be spotted. Most cases showed an air leak coming from a ruptured bleb localized in the apical segment of either the upper or the lower lobe. In one patient the air leak came from a lung tear of about two centimeters near the nodal station n7 (likely an iatrogenic effect of lymph node dissection).

Not only are certain areas within the pleural space more difficult to reach than others, but they may be the specific target to intervene on in order to stop PALs. This need for flexibility and efficacy in a confined space is not answered by a blood patch (unless a large amount of blood is used, with an increased risk of empyema [15] and an intrinsic loss of precision). In our study, no patients receiving FT suffered from postprocedural infection.

FT is relatively painless, with potential mild discomfort at the site of insertion that can easily be addressed by local anesthesia. As a consequence, the patient can cooperate throughout the procedure, i.e., can cough when needed. When ASA scores 1–2 and 3 were combined, no evidence of a difference was found among groups (*p* = 0.124). Nevertheless, an ASA score of 3 was higher in the FT group than in the historical group (41.9% vs. 0%). This shows that it was possible to perform FT even in patients with severe systemic diseases. Indeed, FT is quick and minimally invasive (it is performed through a pre-existing conduit), it requires no learning curve (the surgeon essentially performs VATS with a bronchoscope), it is accurate (the surgeon has a direct and magnified view of the entire pleural space), and it requires only local anesthetic agents (which can be an advantage for frail and elder patients).

Limitations in performing FT could be the patient’s inability to stay still during the procedure (about 20 min) in case of rib fractures or difficulty following instructions, e.g., patients with cognitive impairment.

## 5. Conclusions

To the best of our knowledge, our use of FT to manage PALs after lung resection is the first one reported in the literature. When compared to thoracotomy, no differences were found in terms of time for PAL resolution from aerostasis (*p* = 0.678) and length of in-hospital stay (*p* = 1). Due to its efficacy, increased visual accuracy and minimal discomfort we think that FT can be a novel valid alternative to surgical treatment of PALs via thoracotomy.

The findings of this study have to be seen in the light of some limitations. Data were collected retrospectively with, therefore, no control for potential confounding factors. In addition, the sample size is small and this reduces the possibility to identify slight differences between the groups. Studies with a prospective design including a greater number of patients to overall increase the statistical power, will help to strengthen our results.

## Figures and Tables

**Table 1:** Clinicopathological characteristic of patients (*n* = sample size, % = percentages, [] = ranges).

Variables			*n* (%)
Procedure		Total	23 (100)
		Flexible Thoracoscopy Thoracotomy	17 (74) 6 (26)
Gender		Male	20 (87)
		Female	3 (13)
Smoking status		No	10 (44)
		Former	7 (30)
		Yes	6 (26)
Histology		Adenocarcinoma	12 (52)
		Other	7 (31)
		Squamous cell carcinoma	4 (17)
	
Lung resection	Side	Right	17 (74)
		Left	6 (26)
	Lobe	Upper	14 (61)
		Lower	9 (39)
	Approach	Open	18 (78)
		VATS	5 (22)
Length of in-hospital stay (days), median			24 [10–85]
BMI, median			24 [19–27]
Age at surgery (years), median			70 [22–82]

**Table 2:** Comparison of baseline characteristics between groups (*n* = sample size, % = percentages, [] = ranges).

Variables			Study Groups *n* (%)		*p*-Value
			FT 17 (74)	Thoracotomy 6 (26)	
Gender		Male	14 (82)	6 (100)	0.539
		Female	3 (18)	0 (0)	
Smoking status		Never	7 (41)	3 (50)	0.132
		Former	7 (41)	0 (0)	
		Current	3 (18)	3 (50)	
ASA Score		I-II	10 (59)	6 (100)	0.124
		III	7 (41)	0 (0)	
Comorbidities		Yes	12 (71)	3 (50)	0.621
		No	5 (29)	3 (50)	
Lung resection	Side	Right	14 (82)	3 (50)	0.279
		Left	3 (18)	3 (50)	
	Lobe	Upper	12 (71)	4 (67)	0.162
		Lower	5 (19)	2 (33)	
	Approach	Open	14 (82)	4 (67)	0.576
		VATS	3 (18)	2 (33)	
FEV1%, median			83 [49–96]	75.5 [65–101]	0.491
BMI, median			24 [19–27]	24 [22–26]	0.916
Age at surgery (years), median			71 [47–82]	67 [22–75]	0.076
Operation time (minutes), median			181 [79–345]	195 [69–235]	0.917

**Table 3:** Postoperative characteristics and outcomes (*n* = sample size, % = percentages, [] = ranges).

Variables	Study Groups *n* (%)		*p*-Value
	FT 17 (74)	Thoracotomy 6 (26)	
Days from lung resection to aerostasis, median	13 [7–41]	13.5 [7–20]	1
Days from aerostasis to chest tube removal, median	10 [2–31]	7 [3–73]	0.678
Length of in-hospital stay (days), median	24 [10–53]	23 [17–85]	1

## Data Availability

Data available on request due to privacy.
